# Dynamic sensitivity analysis of biological systems

**DOI:** 10.1186/1471-2105-9-S12-S17

**Published:** 2008-12-12

**Authors:** Wu Hsiung Wu, Feng Sheng Wang, Maw Shang Chang

**Affiliations:** 1 Department of Computer Science and Information Engineering, National Chung Cheng University, Chiayi 62102, Taiwan; 2 Department of Chemical Engineering, National Chung Cheng University, Chiayi 62102, Taiwan

## Abstract

**Background:**

A mathematical model to understand, predict, control, or even design a real biological system is a central theme in systems biology. A dynamic biological system is always modeled as a nonlinear ordinary differential equation (ODE) system. How to simulate the dynamic behavior and dynamic parameter sensitivities of systems described by ODEs efficiently and accurately is a critical job. In many practical applications, e.g., the fed-batch fermentation systems, the system admissible input (corresponding to independent variables of the system) can be time-dependent. The main difficulty for investigating the dynamic log gains of these systems is the infinite dimension due to the time-dependent input. The classical dynamic sensitivity analysis does not take into account this case for the dynamic log gains.

**Results:**

We present an algorithm with an adaptive step size control that can be used for computing the solution and dynamic sensitivities of an autonomous ODE system simultaneously. Although our algorithm is one of the decouple direct methods in computing dynamic sensitivities of an ODE system, the step size determined by model equations can be used on the computations of the time profile and dynamic sensitivities with moderate accuracy even when sensitivity equations are more stiff than model equations. To show this algorithm can perform the dynamic sensitivity analysis on very stiff ODE systems with moderate accuracy, it is implemented and applied to two sets of chemical reactions: pyrolysis of ethane and oxidation of formaldehyde. The accuracy of this algorithm is demonstrated by comparing the dynamic parameter sensitivities obtained from this new algorithm and from the direct method with Rosenbrock stiff integrator based on the indirect method. The same dynamic sensitivity analysis was performed on an ethanol fed-batch fermentation system with a time-varying feed rate to evaluate the applicability of the algorithm to realistic models with time-dependent admissible input.

**Conclusion:**

By combining the accuracy we show with the efficiency of being a decouple direct method, our algorithm is an excellent method for computing dynamic parameter sensitivities in stiff problems. We extend the scope of classical dynamic sensitivity analysis to the investigation of dynamic log gains of models with time-dependent admissible input.

## Background

A mathematical model to understand, predict, control, or even design a real biological system is a central theme in systems biology. The most often used mathematical models for dynamic biological systems are formulated as nonlinear ordinary differential equations (ODEs). The critical challenges to get an ODE model are structure identification and parameter estimation of the model. To identify the structure and parameters of a dynamic model, the most important and essential job is to find the solution of ODEs efficiently and accurately. This job can be treated by analytical and numerical methods. Analytical methods are limited to certain special forms of ODEs, but numerical methods have no such limitations. There are several numerical methods can be used to solve ODEs [1-3], e.g., Taylor-series methods, modified Euler methods, and Runge-Kutta methods with variable step size control. The Taylor-series method takes more computation time to solve ODEs if the various derivatives are complicated, and the error is difficult to determine for arbitrary functions. The modified Euler method is a special case of a second-order Runge-Kutta method, and is more efficient compared to the Taylor-series method [4]. Fourth-order Runge-Kutta method is the most widely used ODE solver to meet requirements on both efficiency and accuracy. Collocation methods [5] are another common algorithms for solving ODEs and have been used for more than forty years. Wang [6] proposed a modified collocation method to transform ODEs into algebraic equations, and solved them by the Newton-Raphson method or an iteration method with step length restriction. The restricted step size is fixed and computed by trial and error. To overcome this drawback, we propose an adaptive step size control approach based on the Banach fixed point theorem for the modified collocation method in this paper.

There are different types of gains and dynamic sensitivities defined for sensitivity analysis [7-9]. The relative change of a dependent variable in response to a relative change in an independent variable is called a *logarithmic gain*, or a *log gain*. Log gains describe the change of dependent variables due to an environment change and are very useful for the assessment of the robustness and parameter estimation of a model. The change of a dependent variable in response to a change in a parameter is called a *parameter sensitivity*. In contrast to log gains, parameter sensitivities are the change of dependent variables correspond to a structure change in the model. The Biochemical Systems Theory (BST) [10] and Metabolic Control Analysis (MCA) [11-13] have achieved a great success in addressing the sensitivities at a steady state. However, the transient or periodic behavior is the primary interest in many systems (e.g., oscillation systems and fermentation systems that do not have a steady state). In these systems, the parameter sensitivities and log gains change with time, therefore the calculation methods for the steady state responses can not function. Dynamic sensitivity analysis is used in studying time-varying sensitivities in dynamic biological systems. A dynamic biological system can be characterized using logarithmic gains, sensitivities with respect to parameters and initial conditions. Several methods have been published to evaluate dynamic sensitivities [14-24]. They can be divided into the indirect methods (IDMs) and the direct methods (DMs). In the IDMs, the value of one dedicated parameter is varied infinitesimally while the values of other parameters are fixed. The model equations are solved anew for these sets of values of the parameters that differ in the value of the dedicated parameter only. The sensitivity of each variable with respect to this dedicated parameter is computed using the difference between the solutions of that variable for the two sets of values of the parameters divided by the infinitesimal difference of the dedicated parameter. In the DMs, using an ODE solver to solve the model equations and sensitivity equations simultaneously is the most used method for computing dynamic sensitivities. Shiraishi et al. [25] published a variable-order, variable-step Taylor-series method that can be used as an ODE solver providing a highly accurate calculation to compute dynamic sensitivities. This method is limited to general mass action (GMA) models described by power-law differential equations. Runge-Kutta methods with variable step size control can be used to compute dynamic sensitivities for most of the nonlinear differential equations, but is inefficient to determine the step size in a large dimensional system including the model differential equations and sensitivity differential equations. Due to the efficiency, Dunker [15] proposed the decoupled direct method (DDM), in which the sensitivity equations are solved separately from the model equations. He said: "the decoupled method has advantages in simplicity, stability, accuracy, efficiency, storage requirements, and program size over other methods". Although the DDM approach has so many advantages, the step size for the time profile determined by the error control of model equations is unable to be used for the sensitivities when the sensitivity equations are more stiff than the model equations and will generate inaccurate results.

Dynamic sensitivity analysis evaluates the influences on dependent variables due to variations of parameters, initial conditions and independent variables. In many practical applications, e.g., the fed-batch fermentation systems, the system admissible input (corresponding to independent variables of the system) can be time-dependent. The main difficulty for investigating the dynamic log gains of these systems is the infinite dimension due to the time-dependent input. Shiraishi et al. [26] proposed an efficient method, based on a combination of the recasting technique and the Taylor-series method, for calculating the time courses of log gains to investigate the dynamic behavior of log gains for oscillation models with a limit cycle. The method is limited to the computations of dynamic log gains with respect to constant independent variables. We extend the computations of dynamic log gains to a model with continuous time-varying admissible input based on the finite parameterization method (PM). The classical PM was created for numerical solutions of optimal control problems [27]. The central idea of the method relies on a simple approximation mechanism. The whole time domain of a continuous admissible input is partitioned into several subintervals, and the input for each subinterval is approximated by a piecewise constant function. The dynamic log gains with respect to the continuous admissible input can be computed based on the partial derivations of dependent variables with respect to the piecewise constant input [28-30].

In this paper, we present an algorithm with an adaptive step size control that can be used for computing the solution and dynamic sensitivities of an autonomous ODE system simultaneously. This algorithm is the modified collocation method, proposed by Wang [6], with an adaptive step size control approach. Although our algorithm is one of the decouple direct methods in computing dynamic sensitivities of an ODE system, the step size determined by model equations can be used on the computations of the time profile and dynamic sensitivities with moderate accuracy even when sensitivity equations are more stiff than model equations. In the algorithm, the modified collocation method is used to transform model and sensitivity equations into algebraic equations, and the approximated solution is solved by an iteration method. This algorithm can be extended easily to solve problems of mixed differential and algebraic equations (DAEs) by combing algebraic equations with that transferred from differential equations. In our algorithm for computing dynamic sensitivities of an ODE system, the model equations and sensitivity equations are solved alternatively in two stages. First, the model equations are advanced from *t*_*i *_to *t*_*i *_+ *η *using the iteration method, where *η *is the step size decided by model equations based on the fixed-point theorem. Second, the solution of model equations at *t*_*i *_+ *η *and the same step size are used to propagate the sensitivity equations from *t*_*i *_to *t*_*i *_+ *η*. For dynamic systems with continuous time-dependent admissible input, the dynamic log gains are computed based on the parameterization techniques. The PM is used to approximate the original infinite-dimensional problem by a finite dimensional one with piecewise constant input. The dynamic log gain for this approximation problem is defined as the percentage change of a dependent variable in response to an infinitesimal percentage change for each piecewise constant input.

To show this algorithm can perform dynamic sensitivity analysis on very stiff ODE systems with moderate accuracy, it is implemented and applied to two sets of chemical reactions: pyrolysis of ethane and oxidation of formaldehyde. The accuracy of this algorithm is demonstrated by comparing the dynamic parameter sensitivities from this new method and that from the direct method with Rosenbrock stiff integrator based on the indirect method. The same dynamic sensitivity analysis is performed on an ethanol fed-batch fermentation system with a time-varying feed rate to evaluate the applicability of the algorithm to realistic models with time-dependent admissible input.

## Results and discussion

To illustrate the accuracy of our algorithm, it is implemented and applied to stiff chemical mechanisms for the pyrolysis of ethane as well as the oxidation of formaldehyde. These systems have been shown to be unstable using both the DM and the Green's function method [15]. The same dynamic sensitivity analysis is performed on an ethanol fed-batch fermentation system with a time-varying feed rate to evaluate the applicability of the algorithm to realistic models with time-dependent admissible input.

### Pyrolysis of ethane

The chemical mechanism for the pyrolysis of ethane is a very stiff system and consists of seven species in five reactions. The chemical reactions and rate constants are shown in Table 1 and are described by GMA model equations as follows:

$$\begin{array}{c} \frac{\text{d}[CH_{3}]}{\text{d}t}=2k_{1}[C_{2}H_{6}]-k_{2}[CH_{3}][C_{2}H_{6}]\\ \frac{\text{d}[CH_{4}]}{\text{d}t}=k_{2}[CH_{3}][C_{2}H_{6}]\\ \frac{\text{d}[C_{2}H_{4}]}{\text{d}t}=k_{3}[C_{2}H_{5}]\\ \frac{\text{d}[C_{2}H_{5}]}{\text{d}t}=k_{2}[CH_{3}][C_{2}H_{6}]+k_{4}[C_{2}H_{6}][H]-k_{3}[C_{2}H_{5}]\\ \frac{\text{d}[C_{2}H_{6}]}{\text{d}t}=-k_{1}[C_{2}H_{6}]-k_{2}[CH_{3}][C_{2}H_{6}]-k_{4}[C_{2}H_{6}][H]\\ \frac{\text{d}[H]}{\text{d}t}=k_{3}[C_{2}H_{5}]-k_{4}[C_{2}H_{6}][H]-2k_{5}{\left[H\right]}^{2}\\ \frac{\text{d}[H_{2}]}{\text{d}t}=k_{4}[C_{2}H_{6}][H]+k_{5}{\left[H\right]}^{2} \end{array}$$

where [*x*] is the concentration of species *x *and *k*_*i *_is the rate constant. The initial concentration of *C*_2_*H*_6 _is 5.951 × 10^-6 ^mol/cm^3 ^and all other initial concentrations are zeros. All sets of sensitivity coefficients with respect to all rate constants and initial conditions are computed simultaneously without any difficulty using our algorithm with a tolerance of 10^-7^. The normalized sensitivity coefficients for the pyrolysis of ethane at 1 s and 20 s calculated by our algorithm are shown in Table 2. The results obtained by the indirect method (IDM) according to the finite difference approximation and the direct method with Rosenbrock stiff integrator (R/DM) [24] are also given in Table 2 for comparison. The results of our algorithm are of equal accuracy to R/DM in comparison to the IDM and the maximum relative error is 0.58%.

**Table 1:** The mechanism for ethane pyrolysis.

Reaction	Rate constants
*C*_2_*H*_6 _→ *CH*_3 _+ *CH*_3_	1.14 × 10^-2^
*CH*_3 _+ *C*_2_*H*_6 _→ *CH*_4 _+ *C*_2_*H*_5_	1.19 × 10^6^
*C*_2_*H*_5 _→ *C*_2_*H*_4 _+ *H*	1.57 × 10^3^
*H *+ *C*_2_*H*_6 _→ *H*_2 _+ *C*_2_*H*_5_	9.72 × 10^8^
*H *+ *H *→ *H*_2_	6.99 × 10^13^

The initial concentration of *C*_2_*H*_6 _is 5.951 × 10^-6 ^mol/cm^3 ^and all other initial concentrations are zeros. Units for rate constants are mol, cm, s and the temperature is 923 K.

**Table 2:** Sensitivity coefficients for ethane pyrolysis.

Species *x*_*i*_	∂ ln [*x*_*i*_]/∂ *k*_1 _at 1 s			∂ ln [*x*_*i*_]/∂*k*_1 _at 20 s		
		
	IDM	R/DM	our algorithm	IDM	R/DM	our algorithm
*CH*_3_	1.000	1.000	0.99986	1.000	1.000	1.00000
*CH*_4_	0.976	0.976	0.97625	0.644	0.644	0.64350
*C*_2_*H*_4_	0.680	0.680	0.68039	0.324	0.323	0.32348
*C*_2_*H*_5_	0.662	0.662	0.66149	-0.209	-0.210	-0.20950
*C*_2_*H*_6_	-0.044	-0.044	-0.04425	-0.819	-0.819	-0.81896
*H*	0.478	0.478	0.47783	0.091	0.091	0.09053
*H*_2_	0.602	0.602	0.60214	0.221	0.221	0.22098

A tolerance of 10^-8 ^is used for R/DM. A fourth-order adaptive Rosenbrock algorithm is used for integration and ± 5% variations of *k*_1 _are used in the computation of the sensitivities w.r.t. *k*_1 _using indirect method.

### Oxidation of formaldehyde

The formaldehyde oxidation mechanism is a larger system, involves 15 species in 25 reactions. The chemical reactions and rate constants are shown in Table 3 and are described by GMA model equations as follows:

$$\begin{array}{c} \frac{\text{d}[HCO]}{\text{d}t}=k_{2}[HO_{2}][CH_{2}O]+k_{4}[CH_{2}O][OH]+k_{11}[CH_{2}O][H]+k_{16}[CH_{2}O][O]\\ +k_{22}[O_{2}][CH_{2}O]-k_{1}[HCO][O_{2}]-k_{20}[HCO]\\ \frac{\text{d}[O_{2}]}{\text{d}t}=k_{8}{\left[HO_{2}\right]}^{2}+k_{14}[HO_{2}][M]+k_{25}[HO_{2}][H]\\ -k_{1}[HCO][O_{2}]-k_{12}[O_{2}][H]-k_{13}[O_{2}][M][H]-k_{22}[O_{2}][CH_{2}O]\\ \frac{\text{d}[HO_{2}]}{\text{d}t}=k_{1}[HCO][O_{2}]+k_{5}[H_{2}O_{2}][OH]+k_{13}[O_{2}][M][H]+k_{17}[H_{2}O_{2}][H]\\ +k_{19}[H_{2}O_{2}][O]+k_{22}[O_{2}][CH_{2}O]-k_{2}[HO_{2}][CH_{2}O]-k_{7}[HO_{2}]\\ -2k_{8}{\left[HO_{2}\right]}^{2}-k_{10}[HO_{2}][CO]-k_{14}[HO_{2}][M]-(k_{23}+k_{24}+k_{25})[HO_{2}][H]\\ \frac{\text{d}[CO]}{\text{d}t}=k_{1}[HCO][O_{2}]+k_{20}[HCO]-k_{9}[CO][OH]-k_{10}[HO_{2}][CO]\\ \frac{\text{d}[CH_{2}O]}{\text{d}t}=-k_{2}[HO_{2}][CH_{2}O]-k_{4}[CH_{2}O][OH]-k_{11}[CH_{2}O][H]-k_{16}[CH_{2}O][O]\\ -k_{22}[O_{2}][CH_{2}O]\\ \frac{\text{d}[H_{2}O_{2}]}{\text{d}t}=k_{2}[HO_{2}][CH_{2}O]+k_{8}{\left[HO_{2}\right]}^{2}-k_{3}[H_{2}O_{2}][M]-k_{5}[H_{2}O_{2}][OH]\\ -k_{6}[H_{2}O_{2}]-(k_{17}+k_{18})[H_{2}O_{2}][H]-k_{19}[H_{2}O_{2}][O]\\ \frac{\text{d}[M]}{\text{d}t}=0\\ \frac{\text{d}[OH]}{\text{d}t}=2k_{3}[H_{2}O_{2}][M]+k_{10}[HO_{2}][CO]+k_{12}[O_{2}][H]+k_{15}[H_{2}][O]\\ +k_{16}[CH_{2}O][O]+k_{18}[H_{2}O_{2}][H]+k_{19}[H_{2}O_{2}][O]+2k_{23}[HO_{2}][H]\\ -k_{4}[CH_{2}O][OH]-k_{5}[H_{2}O_{2}][OH]-k_{9}[CO][OH]-k_{21}[OH][H_{2}]\\ \frac{\text{d}[H_{2}O]}{\text{d}t}=k_{4}[CH_{2}O][OH]+k_{5}[H_{2}O_{2}][OH]+k_{18}[H_{2}O_{2}][H]+k_{21}[OH][H_{2}]\\ +k_{24}[HO_{2}][H]\\ \frac{\text{d}[H_{2}O_{2}(wall)]}{\text{d}t}=k_{6}[H_{2}O_{2}]\\ \frac{\text{d}[HO_{2}(wall)]}{\text{d}t}=k_{7}[HO_{2}]\\ \frac{\text{d}[CO_{2}]}{\text{d}t}=k_{9}[CO][OH]+k_{10}[HO_{2}][CO]\\ \frac{\text{d}[H]}{\text{d}t}=k_{9}[CO][OH]+k_{14}[HO_{2}][M]+k_{15}[H_{2}][O]+k_{20}[HCO]\\ +k_{21}[OH][H_{2}]-k_{11}[CH_{2}O][H]-k_{12}[O_{2}][H]-k_{13}[O_{2}][M][H]\\ -(k_{17}+k_{18})[H_{2}O_{2}][H]-(k_{23}+k_{24}+k_{25})[HO_{2}][H]\\ \frac{\text{d}[H_{2}]}{\text{d}t}=k_{11}[CH_{2}O][H]+k_{17}[H_{2}O_{2}][H]+k_{25}[HO_{2}][H]-k_{15}[H_{2}][O]\\ -k_{21}[OH][H_{2}]\\ \frac{\text{d}[O]}{\text{d}t}=k_{12}[O_{2}][H]+k_{24}[HO_{2}][H]-k_{15}[H_{2}][O]-k_{16}[CH_{2}O][O]\\ -k_{19}[H_{2}O_{2}][O] \end{array}$$

where [*x*] is the concentration of species *x *and *k*_*i *_is the rate constant. The initial concentrations in mol/cm^3 ^are [*CH*_2_*O*] = 1.124 × 10^-7^, [*O*_2_] = 2.109 × 10^-6^, [*CO*] = 4.699 × 10^-6^, [*M *] = 1.1772 × 10^-5^, and all other initial concentrations are zeros. Sensitivity coefficients with respect to all rate constants and initial conditions are computed successfully using our algorithm with a tolerance of 10^-9^. The normalized sensitivity coefficients for *O *and *H*_2_*O *at 0.005 s calculated by our algorithm are presented in Table 4. The results obtained by IDM and the direct method with Rosenbrock stiff integrator (R/DM) are also given in Table 4 for comparison. Our results are in good agreement with the R/DM in comparison to the IDM, and the maximum relative error is 0.25%. The discrepancies between the results of our algorithm and the R/DM method are sufficiently small to prove that this new method is capable of performing dynamic sensitivity analysis for stiff differential equations as accurate as direct methods.

**Table 3:** The mechanism for formaldehyde oxidation.

Reaction	Rate constants
*HCO *+ *O*_2 _→ *HO*_2 _+ *CO*	6.02 × 10^10^
*HO*_2 _+ *CH*_2_*O *→ *H*_2_*O*_2 _+ *HCO*	3.43 × 10^10^
*H*_2_*O*_2 _+ *M *→ 2*OH *+ *M*	4.01 × 10^6^
*OH *+ *CH*_2_*O *→ *H*_2_*O *+ *HCO*	9.64 × 10^13^
*OH *+ *H*_2_*O*_2 _→ *H*_2_*O *+ *HO*_2_	3.07 × 10^12^
*H*_2_*O*_2 _→ *H*_2_*O*_2_(*wall*)	1.05 × 10^2^
*HO*_2 _→ *HO*_2_(*wall*)	1.05 × 10^1^
*HO*_2 _+ *HO*_2 _→ *H*_2_*O*_2 _+ *O*_2_	1.81 × 10^12^
*OH *+ *CO *→ *CO*_2 _+ *H*	1.99 × 10^11^
*HO*_2 _+ *CO *→ *CO*_2 _+ *OH*	7.23 × 10^8^
*H *+ *CH*_2_*O *→ *H*_2 _+ *HCO*	1.63 × 10^12^
*H *+ *O*_2 _→ *OH *+ *O*	3.32 × 10^10^
*H *+ *O*_2 _+ *M *→ *HO*_2 _+ *M*	3.63 × 10^15^
*HO*_2 _+ *M *→ *H *+ *O*_2 _+ *M*	2.83 × 10^5^
*O *+ *H*_2 _→ *OH *+ *H*	1.82 × 10^11^
*O *+ *CH*_2_*O *→ *OH *+ *HCO*	6.02 × 10^13^
*H *+ *H*_2_*O*_2 _→ *HO*_2 _+ *H*_2_	7.83 × 10^11^
*H *+ *H*_2_*O*_2 _→ *H*_2_*O *+ *OH*	3.55 × 10^12^
*O *+ *H*_2_*O*_2 _→ *OH *+ *HO*_2_	6.02 × 10^10^
*HCO *→ *H *+ *CO*	4.60 × 10^-12^
*OH *+ *H*_2 _→ *H*_2_*O *+ *H*	6.02 × 10^12^
*CH*_2_*O *+ *O*_2 _→ *HCO *+ *HO*_2_	1.75 × 10^4^
*H *+ *HO*_2 _→ 2*OH*	3.01 × 10^12^
*H *+ *HO*_2 _→ *H*_2_*O *+ *O*	3.01 × 10^13^
*H *+ *HO*_2 _→ *H*_2 _+ *O*_2_	2.71 × 10^13^

The initial concentrations in mol/cm^3 ^are [*CH*_2_*O*] = 1.124 × 10^-7^, [*O*_2_] = 2.109 × 10^-6^, [*CO*] = 4.699 × 10^-6^, [*M*] = 1.1772 × 10^-5 ^and all other initial concentrations are zeros. Units for rate constants are mol, cm, s and the temperature is 952 K.

**Table 4:** Sensitivity coefficients for formaldehyde oxidation.

Rate constant	∂ ln [*HO*_2_]/∂*k*_*i*_			∂ ln [O]/∂*k*_*i*_		
		
	IDM	R/DM	our algorithm	IDM	R/DM	our algorithm
*k*_2_	0.683	0.683	0.68255	0.827	0.827	0.82719
*k*_3_	0.700	0.700	0.69986	0.835	0.835	0.83486
*k*_4_	-0.210	-0.209	-0.20917	-1.160	-1.156	-1.15579
*k*_8_	-0.306	-0.306	-0.30569	-0.296	-0.296	-0.29599
*k*_9_	0.210	0.210	0.20962	1.156	1.156	1.15628
*k*_10_	0.164	0.164	0.16373	1.031	1.031	1.03065
*k*_11_	-0.121	-0.121	-0.12087	-0.660	-0.659	-0.65906
*k*_12_	0.188	0.188	0.18848	0.979	0.979	0.97926
*k*_13_				-0.327	-0.327	-0.32713
*k*_16_				-1.002	-1.000	-0.99990
*k*_22_	0.686	0.685	0.68536	0.742	0.742	0.74169

A tolerance of 10^-15 ^is used for R/DM. A fourth-order adaptive Rosenbrock algorithm is used for integration and ± 5% variations of *k*_*i *_are used in the computation of the sensitivities w.r.t. *k*_*i *_using indirect method.

### Ethanol fed-batch fermentation

The dynamic sensitivity analysis of an ethanol fed-batch fermentation process, a real dynamic biological system never reaching a steady state, is used to elucidate the applicability of our algorithm. Wang et al. [31] built a mathematical kinetic model of fermentation for ethanol and glycerol production using *Saccharomyces diastaticus *LORRE 316, which is a high ethanol tolerance yeast. The mathematical kinetic model for the fed-batch process consists of the dynamic behavior of biomass, glucose, ethanol and glycerol, and its dynamic mass balance equations are expressed as follows:

$$\begin{array}{c} \frac{\text{d}x}{\text{d}\tau }=t_{f}\left(\mu x-\frac{F}{V}x\right),\\ \frac{\text{d}s}{\text{d}\tau }=t_{f}\left[-\left(\frac{q_{p_{1}}}{Y_{p_{1}/s}}+\frac{q_{p_{2}}}{Y_{p_{2}/s}}\right)x+\frac{F}{V}\left(s_{F}-s\right)\right],\\ \frac{\text{d}p_{1}}{\text{d}\tau }=t_{f}\left(q_{p_{1}}x-\frac{F}{V}p_{1}\right),\\ \frac{\text{d}p_{2}}{\text{d}\tau }=t_{f}\left(q_{p_{2}}x-\frac{F}{V}p_{2}\right),\\ \frac{\text{d}V}{\text{d}\tau }=t_{f}F, \end{array}$$

where *x *is the concentration of cell mass, *s *is the concentration of glucose, *p*_1 _is the concentration of ethanol, *p*_2 _is the concentration of glycerol, *V *is the working volume of the fermenter, *t*_*f *_is the final fermentation time, *τ *= *t*/*t*_*f *_is the normalized fermentation time, *s*_*F *_is the feed concentration of glucose, *F *is the feed rate, $Y_{p_{1}/s}$ is the ethanol yield factor, and $Y_{p_{2}/s}$ is the glycerol yield factor. The unstructured kinetic models for the specific cell growth and product formation are respectively expressed as follows:

$$\begin{array}{c} \mu =\frac{\mu_{m}s}{K_{s}+s+s^{2}/K_{s_{I}}}\frac{K_{p_{1}}}{K_{p_{1}}+p_{1}+p_{1}^{2}/K_{p_{1_{I}}}}\frac{K_{p_{2}}}{K_{p_{2}}+p_{2}+p_{2}^{2}/K_{p_{2_{I}}}},\\ q_{p_{1}}=\frac{\nu_{p_{1}}s}{{K^{\prime }}_{s}+s+s^{2}/{K^{\prime }}_{s_{I}}}\frac{{K^{\prime }}_{p_{1}}}{{K^{\prime }}_{p_{1}}+p_{1}+p_{1}^{2}/K_{{p^{\prime }}_{1I}}},\\ q_{p_{2}}=\frac{\nu_{p_{2}}s}{{K^{\prime \prime }}_{s}+s+s^{2}/{K^{\prime \prime }}_{s_{I}}}\frac{{K^{\prime }}_{p_{2}}}{{K^{\prime }}_{p_{2}}+p_{2}+p_{2}^{2}/{K^{\prime }}_{p_{2I}}}. \end{array}$$

Using a batch fermentation model, Wang et al. obtained the optimal values of 19 parameters [31]. The initial and feed concentrations of glucose are set to 10 and 200 g/L, the initial concentration of biomass is set to 2 g/L, and the starting working volume is set to 1.5 L in the computations of optimal feed rate and optimal fermentation time to maximize the ethanol production rate *J *= *p*_1_*V/t*_*f *_under some physical constraints, e.g., the residual glucose restriction *s*(*t*_*f*_) ≤ *s*_*r *_for reducing the separation cost, *s*_*r *_is the concentration of the desired residual glucose. The optimal final fermentation time is 12.836 hours and the optimal feed rate F* for the fed-batch fermentation model [31] is as follows:

(1) $$F^{*}=\{\begin{array}{ll} 1.0 & \text{for }0\leq \tau \leq 0.1\\ 0.8067 & \text{for }0.1<\tau \leq 0.2\\ 0.5567 & \text{for }0.2<\tau \leq 0.3\\ 0.3067 & \text{for }0.3<\tau \leq 0.4\\ 0.0557 & \text{for }0.4<\tau \leq 0.5\\ 1.3687\times 10^{-18} & \text{for }0.5<\tau \leq 0.6\\ 6.2749\times 10^{-18} & \text{for }0.6<\tau \leq 0.7\\ 0.0 & \text{for }0.7<\tau \leq 0.8\\ 0.0 & \text{for }0.8<\tau \leq 0.9\\ 2.1883\times 10^{-20} & \text{for }0.9<\tau \leq 1 \end{array}$$

**Figure 1:**
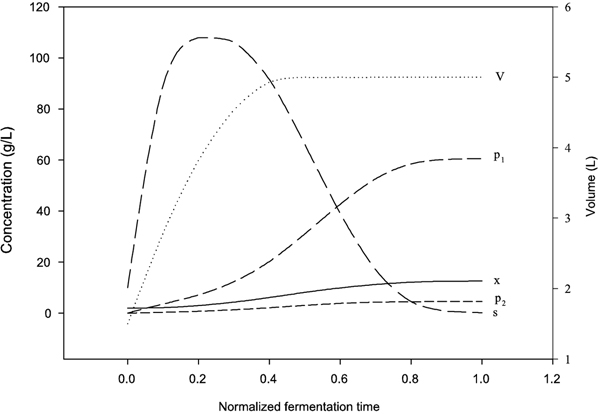
**Dynamic behavior of the ethanol fed-batch fermentation model**. Time profiles of cell mass (*x*), glucose (*s*), ethanol (*p*_1_), glycerol (*p*_2_), and the working volume (*V*) for the ethanol fed-batch fermentation computed using the optimal feed rate F* and the feed glucose of 200 g/L. The horizontal scale is in normalized fermentation time (*t*/*t*_*f*_).

Figure 1 shows the computational time profile of the ethanol fed-batch fermentation model with the optimal feed rate.

**Figure 2:**
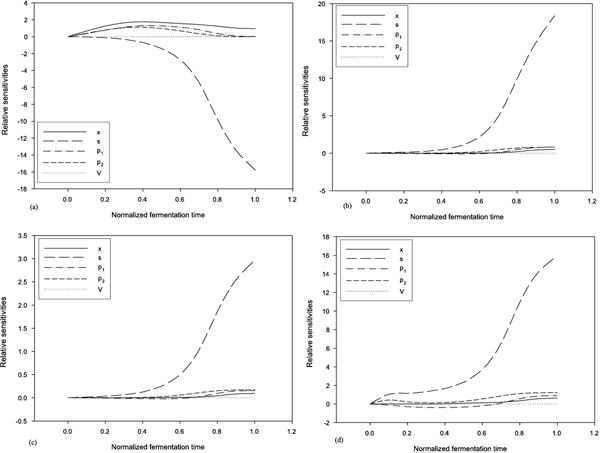
**Dynamic relative sensitivities with respect to *μ*_*m*_**, $Y_{p_{1}/s}$, $Y_{p_{2}/s}$**, and *s*_*F*_**. (a) relative sensitivities with respect to *μ*_*m*_; (b) relative sensitivities with respect to $Y_{p_{1}/s}$; (c) relative sensitivities with respect to $Y_{p_{2}/s}$; (d) relative sensitivities with respect to *s*_*F*_. The horiziontal scale is in normalized fermentation time (*t*/*t*_*f*_).

Our algorithm is applied to the ethanol fed-batch fermentation model using the initial conditions as described above. All dynamic sensitivities with respect to 22 parameters (including *s*_*F*_, *F *and *t*_*f*_) and initial conditions, and the dynamic log gains with respect to time-varying feed rate are computed simultaneously without any difficulty. Figure 2 shows the dynamic relative sensitivities with respect to *μ*_*m*_, $Y_{p_{1}/s}$, $Y_{p_{2}/s}$, and *s*_*F*_. When the maximum specific growth rate *μ*_*m *_is increasing, the rate of consuming glucose is increasing such that the concentration of residue glucose is decreasing. This situation is compatible with the trend in Figure 2(a). The increases in the ethanol and glycerol yield factor cause the increases in the production of ethanol and glycerol, and more glucose remains at the final time. As Figures 2(b) and 2(c) show, to increase the production of ethanol and glycerol by improving the ethanol yield factor is better than by increasing the glyverol yield factor. Figure 2(d) shows that if the feed concentration of glucose is increasing, the cell growth and the production of ethanol and glycerol are increasing. Under this condition, *S. diastaticus *LORRE 316 is unable to completely consume glucose to produce ethanol during the fermentation time and more glucose remains at the final time.

**Figure 3:**
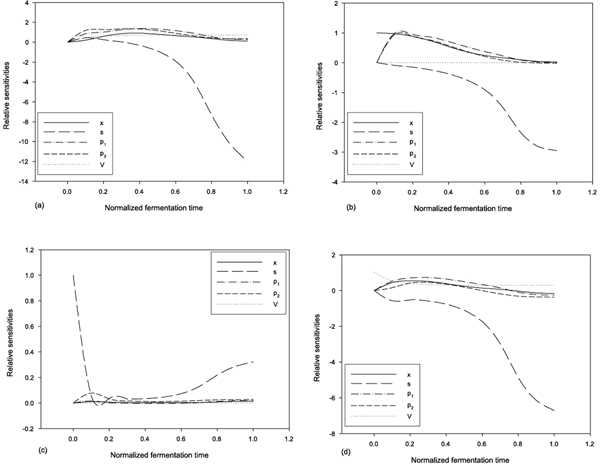
**Dynamic relative sensitivities with respect to *t*_*f*_, *x*(0), *s*(0), and *V*(0)**. (a) relative sensitivities with respect to *t*_*f*_; (b) relative sensitivities with respect to the initial value of *x*; (c) relative sensitivities with respect to the initial value of *s*; (d) relative sensitivities with respect to the initial value of *V*. The horizontal scale is in normalized fermentation time (*t*/*t*_*f*_).

The relative sensitivity with respect to *t*_*f *_is shown in Figure 3(a). As expected, an increase in *t*_*f *_causes a low relative increase in the concentration of cell mass and a high relative decrease in the concentration of residue glucose. Figures 3(b), 3(c) and 3(d) show the dynamic relative sensitivities with respect to the initial conditions *x*, *s, *and *V*. When the initial concentration of cell mass increases, the residue glucose decreases, and the production of ethanol will increase a little, but the production of glycerol will decrease a little at the final fermentation time. Starting the fermentation process with more glucose will cause more glucose to remain and the production of ethanol and glycerol to increase a little at the final fermentation time as shown in Figure 3(c). Figure 3(d) shows that if the initial working volume is increasing, all concentrations of cell mass, glucose, ethanol, and glycerol are decreasing at the final fermentation time.

**Figure 4:**
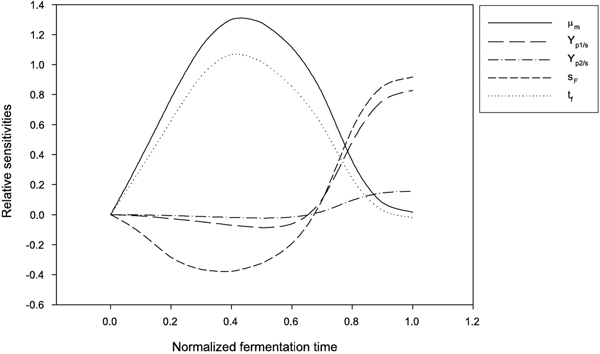
**Dynamic relative sensitivities of *J***. The relative sensitivities of ethanol production rate with respect to *μ*_*m*_, $Y_{p_{1}/s}$, $Y_{p_{2}/s}$, *s*_*F*_, and *t*_*f*_. The horizontal scale is in normalized fermentation time (*t*/*t*_*f*_).

We are interested in the ethanol production rate *J *in the fermentation process. The effects on *J *with respect to *μ*_*m*_, $Y_{p_{1}/s}$, $Y_{p_{2}/s}$, *s*_*F*_, and *t*_*f *_ are shown in Figure 4. To increase *J*, it is clear that an increase in $Y_{p_{1}/s}$ or *s*_*F *_will have more impact than an equal relative increase in $Y_{p_{2}/s}$ or *μ*_*m*_. The negative value of relative sensitivity for *J *with respect to *t*_*f *_means a decrease in the fermentation time will get a higher *J *at the expense of more residual glucose. Though the relative sensitivity of *J *with respect to *s*_*F *_is higher than that with respect to $Y_{p_{1}/s}$ at the final fermentation time, by increasing *s*_*F *_to increase *J *will cause more glucose left at the final time and increase the cost to separate the residue glucose and the ethanol product. We can make a conclusion that to increase *J *by increasing $Y_{p_{1}/s}$ will be a better choice than increasing *s*_*F *_or $Y_{p_{2}/s}$, and decreasing the fermentation time.

The feed rate *F*(*t*) of the fed-batch fermentation model is a time-dependent input control variable, so that the computation of the effect on *J *with respect to *F*(*t*) is an infinite dimensional problem. The fermentation time is divided into ten equal time partitions, and the optimal feed rate F* for the fed-batch fermentation model [31] is approximated by ten piecewise constant functions.  The ten input control parameters, denoted by *F*_*i*_, *i *= 1,..., 10, are shown in equation (1). The proposed algorithm computes the dynamic log gains based on the parameterization method. All dynamic log gains with respect to *F*_*i*_, *i *= 1,..., 10, are computed (data not shown here) with the parameter sensitivities simultaneously. The dynamic log gains of *J *with respect to *F*_*i*_, *i *= 1,..., 10, are computed by

$$\frac{\partial \ln J}{\partial \ln F_{i}}=\frac{\partial \ln p_{1}}{\partial \ln F_{i}}+\frac{\partial \ln V}{\partial \ln F_{i}}.$$

**Figure 5:**
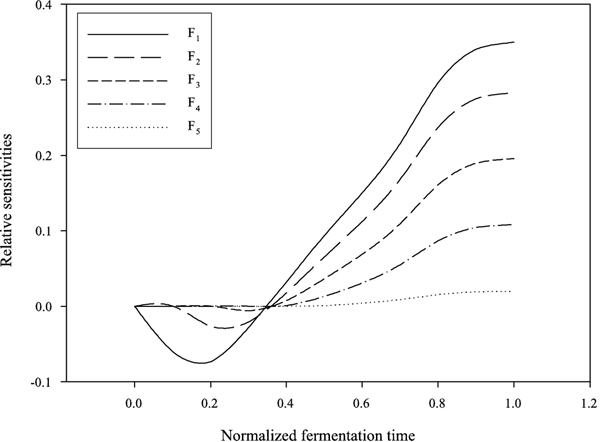
**Dynamic log gains of *J *w.r.t. *F*(*t*)**. The dynamic log gains of ethanol production rate with respect to *F*_*i*_, *i *= 1,..., 5. The horizontal scale is in normalized fermentation time (*t*/*t*_*f*_).

Due to the optimal values of *F*_*i*_, *i *= 6,..., 10 are equal to or very close to 0, The dynamic log gains of *J *with respect to them are small and can be ignored. The dynamic log gains of *J *with respect to *F*_*i*_, *i *= 1,..., 5, are shown in Figure 5. The effects on *J *are in decreasing order from *F*_1 _to *F*_5_. Increasing the feed rate at an early stage will get a higher *J *at the final fermentation time than that at a later stage without considering the residual glucose.

## Conclusion

To deeply study the dynamic behavior of a biological system, one of the methods is to model it as a mathematical model. The most used mathematical model for simulating biological systems is the ODE model. The essential task for modeling and simulating a biological system is to find the solution of an ODE model efficiently and accurately. We present an algorithm with an adaptive step size control that can be used for computing the solution and dynamic sensitivities of an ODE system simultaneously. Instead of using error control to decide the step size in solving the model equations, our algorithm computes the step size based on the fixed-point theorem and the same step size can be used in solving the sensitivity equations.

Dynamic sensitivity analysis is a useful tool to investigate the behavior of dynamic systems. In the direct methods for solving the dynamic sensitivities, sensitivity equations and model equations are coupled and solved together at the expense of more computation time. In contrast, sensitivity equations and model equations are solved separately in the decouple direct methods. The DDMs are more efficient than the DMs due to the dimension of ODEs. The chief disadvantage of DDMs is the requirement of error control on both model equations and sensitivity equations. Our algorithm with an efficient step control approach based on the fixed-point theorem is used to address the disadvantage of DDMs. Analogous to the DMs, the same step size obtained by model equations is used on both model and sensitivity equations. It has been implemented and applied to well-known stiff problems with the same accuracy compared to the direct method with Rosenbrock stiff integrator (R/DM). As our algorithm is one of the DDMs, it has the efficiency of the DDMs and the same accuracy of the DMs as presented in the section describing the results. By combining the efficiency and accuracy, our algorithm is an excellent method for computing dynamic parameter sensitivities in stiff problems.

We extend the scope of classical dynamic sensitivity analysis to the investigation of dynamic log gains of models with time-dependent admissible input. The parameterization method is used to approximate the infinite-dimensional computation problem for dynamic log gains in models with time-dependent admissible input by a classical finite-dimensional computation problem of dynamic log gains. Then, all dynamic log gains and parameter sensitivities can be obtained simultaneously from our algorithm. Appropriate parameterization allows one to obtain a more efficient way to compute the dynamic log gains with respect to a continuous time-dependent input than that by finite difference approximation. Finally, the new proposed algorithm is applied to the ethanol fed-batch fermentation system, a real dynamic biological system which never reaches a steady state, with a time-varying feed rate for elucidating the applicability to realistic models with time-dependent admissible input. Through the dynamic sensitivity analysis of the ethanol fed-batch fermentation model, we conclude that to get a higher ethanol production rate by increasing the ethanol yield factor is a good choice.

## Methods

A dynamic biological system is always modeled as a nonlinear ODE system:

(2) $$\frac{\text{d}x}{\text{d}t}=Nv(x(t),y(t);\theta ),x(0)=x_{0},y(0)=y_{0},$$

where **x**(*t*) ∈ ℝ^*n *^is a vector of dependent variables, **y**(*t*) ∈ ℝ^*m *^is a vector of independent variables, *θ *∈ ℝ^*p *^is a vector of parameters, **v **∈ ℝ^*q *^is a vector of fluxes between the variables, *N *∈ ℝ^(*n*+*m*) × *q *^is the stoichiometric matrix describing the interconnecting fluxes, **x**_0 _and **y**_0 _are initial concentrations of **x **and **y **respectively. Using a different kinetic description for **v **results in a different mathematical model. In the Michaelis-Menten model, each element *v*_*i *_of **v **is of the form

(3) $$v_{i}=\frac{V_{i}^{max}x_{j}}{K_{i}+x_{j}},i=1,...,q,$$

where *x*_*j *_is the substrate, $V_{i}^{max}$ is the maximum flux for *v*_*i*_, and *K*_*i *_is the half-saturated flux for *v*_*i*_. For the GMA systems, the kinetic equation for *v*_*i *_is expressed as a power-law function

(4) $$v_{i}=\gamma_{i}\prod_{j=1}^{n+m}x_{j}^{g_{i,j}},i=1,...,q.$$

where *x*_*j *_∈ **x **for *j *≤ *n*, *x*_*j *_∈ **y **for *j *> *n*, *γ*_*i *_is the rate constant, and *g*_*ij *_is the kinetic order for each *x*_*j*_. Equation (2) can be expressed concisely as:

(5) $$\frac{\text{d}x}{\text{d}t}=f(x(t),y(t);\theta ),x(0)=x_{0},y(0)=y_{0},$$

where the function **f **is assumed to be continuous and differentiable in all its arguments **x**, **y**,and *θ*. This assumption on **f **is satisfied for both of equations (3) and (4).

For a model described by a nonautonomous dynamic system as follows:

(6) $$\frac{\text{d}x}{\text{d}t}=f(x(t),y(t),t;\theta ),x(0)=x_{0},y(0)=y_{0},$$

we let *x*_*n*+1 _= *t *and *dx*_*n*+1_/*dt *= 1, equation (6) can be rewritten as equation (5) with **x**(*t*) ∈ ℝ^*n*+1^. This is an (*n *+ 1)-dimensional autonomous dynamic system. Similarly, an *n*-dimensional time dependent equation is a special case of an (*n*+ 1)-dimensional autonomous dynamic system. Using this trick, we can always remove any time dependence by adding an extra dimension to the system. Thus, without loss of generality, we will consider the autonomous dynamic systems expressed by equation (5) unless stated otherwise.

### ODE solver

Given a set of ODEs expressed as equation (5) and a set of time points *T *= {*t*_*i*_|*i *= 1,..., *k*}. An ODE solver is to find the value of **x**(*t*_*i*_), *t*_*i *_∈ *T *for a given *θ*. Many ODE solvers with variable step size control can be used to solve equation (5). Wang [6] proposed a modified collocation method with Lagrange polynomials as shape functions to transform ODEs into algebraic equations. The whole time domain of the problem is divided into a number of non-overlapping intervals [*t*_*i*-1_, *t*_*i*_], *i *= 1,..., *k*. The unified formulas of the modified collocation method for the subinterval [*t*_*j*-1_, *t*_*j*_], *t*_*i*-1 _≤ *t*_*j*-1 _<*t*_*j *_≤ *t*_*i*_, can be expressed as

(7) $$x(t_{j})=\hat{I}x(t_{j-1})+\eta_{j}\{Df(x(t_{j}),y(t_{j});\theta )+\hat{D}f(x(t_{j-1}),y(t_{j-1});\theta )\},$$

where *η*_*j *_is the step size in time *t*_*j*_, **Î **is an identity-like matrix, and the coefficient matrices **D **and $\hat{D}$ depend on the shape functions. The accuracy and efficiency of collocation methods depend largely on the degree of shape functions. The modified collocated equations with piecewise linear polynomials transformed from equation (5) for each subinterval [*t*_*j*-1_, *t*_*j*_], *t*_*i*-1 _≤ *t*_*j*-1 _<*t*_*j *_≤ *t*_*i *_have the same formulas as the modified Euler method:

(8) $$x(t_{j})=x(t_{j-1})+\frac{1}{2}\eta_{j}\{f(x(t_{j}),y(t_{j});\theta )+f(x(t_{j-1}),y(t_{j-1});\theta )\}.$$

Instead of solving the ODEs in equation (5) directly, we find the solution of algebraic equations in equation (8) step-by-step for each time interval [*t*_*i*-1_, *t*_*i*_], *i *= 1,..., *k*. The solution obtained from equation (8) is a good approximation solution of ODEs in equation (5) when the step size *η*_*j *_is small enough. How to decide the step size is an important problem for all ODE solvers. A larger step size can cause the solution to be inaccurate and divergent, and a smaller step size is inefficient for the computations. Wang [6] uses the Newton-Raphson method with step length restriction to solve equation (8). The restricted step size is fixed and computed by trial and error. To overcome this drawback, we propose an adaptive step size control approach based on the Banach fixed point theorem for the modified collocation method in this paper. We will show that this approach can determine the step size automatically and efficiently when computing the solutions and dynamic sensitivities of equation (5) simultaneously.

The Banach fixed point theorem and some terminologies for describing the theorem are defined below.

**Definition 1**. Metric space [32]

A metric space (*X*, *d*) is a set *X *where a notion of distance *d *(called a metric) between elements of the set is defined.

**Definition 2**. Cauchy sequence [32]

A sequence (*x*_*n*_) in a metric space (*X*, *d*) is said to be Cauchy if for every *ε *> 0 there is a positive integer *N *such that for all natural numbers *m*, *n *> *N*, the distance *d*(*x*_*m*_, *x*_*n*_) is less than *ε*.

**Definition 3**. Complete metric space [32]

A metric space (*X*, *d*) in which every Cauchy sequence has a limit in *X *is called complete.

**Theorem 4**. *Banach fixed point theorem *[32]

*Let *(*X*, *d*) *be a non-empty complete metric space. A mapping ψ*: *X *→ *X is called a contraction on X if there is a nonnegative real number q *< 1 *such that for all a*, *b in X*

(9) *d*(*ψ*(*a*), *ψ*(*b*)) ≤ *q*·*d*(*a*, *b*).

The contraction ψ on X admits one and only one fixed point x* ∈ *X such that ω *(x*) = x*.

We now show how to decide the step size *η*_*j *_in equation (8) based on the Banach fixed point theorem. Equation (8) is an implicit expression of **x**, and it can be rewritten as

(10) $$\begin{array}{c} x(t_{j})=\frac{1}{2}\eta_{j}f(x(t_{j}),y(t_{j});\theta )+c\\ =g(x(t_{j}),y(t_{j});\theta ), \end{array}$$

where **c **= **x**(*t*_*j*-1_)+0.5*η*_*j*_**f**(**x**(*t*_*j*-1_), **y**(*t*_*j*-1_); *θ*) is a constant vector. When the values of independent variable **y **and *θ *are given, the solution of **x **= **g**(**x**) can be found by an iteration process. A sequence of values of **x**(*t*_*j*_) is obtained using the iterative rule. If **x**^*i*^(*t*_*j*_) tends to a limit x*(*t*_*j*_) when *i *→ ∞, it is the answer of **x **= **g**(**x**) and called a fixed point of the function **g**(**x**). Let *X *be the set of **x**^*i*^(*t*_*j*_), *i *= 1,..., ∞ and *d*(**a**, **b**) = ||**a **- **b**||_*p *_where **a **and **b **are arbitrary **x**^*i*^(*t*_*j*_) and ||·||_*p *_is the *p*-norm. Then (*X*, *d*) forms a non-empty complete metric space. If **g **is a contraction on *X*, the Banach fixed point theorem guarantees the existence of a fixed point and the convergence of the iteration process to that fixed point. By the equation (9) and the definition of distance function *d*, for **a**, **b **∈ *X *we obtain

(11) ||**g**(**a**) - **g**(**b**)||_*p *_≤ *q*||**a **- **b**||_*p*_, *q *< 1.

We suppose that **g**(**x**) is a continuous and differentiable function on *X*. By the generalized mean value theorem and the definition of matrix norm, we have

(12) $$||g(a)-g(b)|{|}_{p}\leq {\left\|\frac{\partial g}{\partial x}\right\|}_{p}||a-b|{|}_{p}.$$

Comparing equations (11) with (12), we obtain

(13) $${\left\|\frac{\partial g}{\partial x}\right\|}_{p}<1,$$

where ||·||_*p *_is the *p*-norm of a matrix. By substitution of the term on the right of the equal sign in equation (10) for **g**, equation (13) can be rewritten as

(14) $$\frac{1}{2}\eta_{j}{\left\|\frac{\partial f(x,y;\theta )}{\partial x}\right\|}_{p}<1.$$

This equation is used to compute the maximum *η*_*j *_with *p *= 2 when the process of finding the solution of equation (8) is in progress. The Jacobian matrix ∂**f**/∂**x **in equation (14) must be evaluated at each time *t*_*j*_. The computation of the Jacobian matrix can be done by evaluating the analytic formula of the partial derivative of **f **with respect to **x **which is user-provided, or by the finite difference approximation. For the GMA systems, the model equations are expressed in power-law and the value of the Jacobian matrix can be straightforwardly obtained using the analytic formula as follows:

$$\frac{\partial f}{\partial x}=N\left[\begin{array}{cccc} g_{11}x_{1}^{-1}v_{1} & g_{12}x_{2}^{-1}v_{1} & \cdots & g_{1n}x_{n}^{-1}v_{1}\\ g_{21}x_{1}^{-1}v_{2} & g_{22}x_{2}^{-1}v_{2} & \cdots & g_{2n}x_{n}^{-1}v_{2}\\ \vdots & \vdots & \ddots & \vdots \\ g_{q1}x_{1}^{-1}v_{q} & g_{q2}x_{2}^{-1}v_{q} & \cdots & g_{qn}x_{n}^{-1}v_{q} \end{array}\right].$$

where *N *is the stoichiometric matrix, *v*_*i *_is the *i*^*th *^element of **v **∈ ℝ^*q *^and *g*_*ij *_is the kinetic order for each *x*_*j *_∈ **x **in *v*_*i*_. For efficiency, we approximate the value of 2-norm of the Jacobian matrix ∂**f**/∂**x **by ||∂**f**/∂**x**||_Δ_, where *n *is the dimension of **x** and ||∂**f**/∂**x**||_Δ _is the maximum absolute value of the element of the Jacobian matrix. The proposed algorithm AMCM, Adaptive Modified Collocation Method, is shown as follows

Algorithm AMCM

**Input**:

1. A set of *n *ordinary differential equations $\dot{x}$ = **f**(**x**, **y**) with *n *dependent variables *x*_*i*_, *i *= 1,..., *n *and *m *independent variables *y*_*i*_, *i *= 1,..., *m*.

2. Two order sets **x**_0 _= {*x*_*i*_(*t*_0_)|*i *= 1,..., *n*} of initial values of **x **and **y**_0 _= {*y*_*i*_(*t*_0_)|*i *= 1,..., *m*} of initial values of **y**.

3. An order set *T *= {*t*_1_,..., *t*_*k*_} of sampling points, *t*_*i*_, 1 ≤ *i *≤ *k *is the sampling time of the solution of each ODE, *k *is the number of sampling points.

4. A tolerance *ε*

**Output**: The set of solutions of dependent variables at each sampling time.

• For each sampling time *t*_*i *_in *T*.

1. *η*_*j *_← *t*_*i *_- *t*_*i*-1_, *d*_*t *_← 0, **x**^*c *^← **x**(*t*_*i*-1_), **y**^*c *^← **y**(*t*_*i*-1_)

2. Repeat the following steps until *d*_*t *_= *t*_*i *_- *t*_*i*-1_.

(a) **x**^*p *^← **x**^*c*^, **y**^*p *^← **y**^*c*^

(b) Evaluate the Jacobin matrix $A\leftarrow \frac{\partial f(x^{p},y^{p})}{\partial x}$

(c) Compute the upper bound *μ *of the value of ||**A**||_2 _by $\sqrt{n(m+n)}||A|{|}_{\Delta },||A|{|}_{\Delta }\equiv \underset{i,j}{\max}|a_{ij}|$

(d) If μ * *ε *≥ 1, it means the ODEs are stiff, then exit this algorithm.

(e) If μ * *η*_*j *_> 1, then update *η*_*j *_with 0.9/*μ*

(f) Call iteration algorithm to compute the value of **x**^*c *^stepped forward *η*_*j *_from **x**^*p*^.

(g) If the iteration algorithm succeeds in computing **x**^*c*^, then *d*_*t *_← *d*_*t *_+ *η*_*j *_and *η*_*j *_← *t*_*i *_- *t*_*i*-1 _- *d*_*t*_, otherwise exist this algorithm.

3. **x**(*t*_*i*_) ← **x**^*c*^, **y**(*t*_*i*_) ← **y**^*c*^.

• return **x**(*t*_*i*_), *i *= 1,..., *k*.

End of Algorithm AMCM

**Algorithm **Iteration

**Input**:

1. A set of *n *ordinary differential equations $\dot{x}$ = **f**(**x**, **y**).

2. **x**(*t*), **y**(*t*), *η*_*j *_and the iteration limitation.

**Output**: **x**(*t *+ *η*_*j*_).

1. Evaluate the value of **f**(**x**(*t*), **y**(*t*)).

2. **x**(*t *+ *η*_*j*_) ← **x**(*t*) + **f**(**x**(*t*), **y**(*t*)) * *η*_*j*_.

3. **y**(*t *+ *η*_*j*_) ← **y**(*t*) + **f**(**x**(*t*), **y**(*t*)) * *η*_*j*_.

4. Repeat the following steps until the iteration limitation is reached or the value of **x**(*t *+ *η*_*t*_) converges.

(a) Evaluate the value of **f**(**x**(*t *+ *η*_*j*_), **y**(*t *+ *η*_*j*_)).

(b) **x**(*t *+ *η*_*j*_) ← **x**(*t*) + 0.5 * *η*_j _* (**f**(**x**(*t*), **y**(*t*)) + **f**(**x**(*t *+ *η*_*j*_), **y**(t + *η*_*j*_)))

5. If the iteration limitation is reached, then exit this algorithm; otherwise, return **x**(*t *+ *η*_*j*_).

**End of Algorithm **Iteration

### Dynamic sensitivity Solver

For a model described by equation (5), the absolute parameter sensitivity *s*(*x*_*i*_, *θ*_*j*_) of dependent variable *x*_*i *_∈ **x **with respect to parameter *θ*_*j *_∈ *θ *is defined as

(15) $$s(x_{i},\theta_{j})=\underset{\Delta \theta_{j}\to 0}{\lim}\frac{x_{i}(t;\theta_{j}+\Delta \theta_{j})-x_{i}(t;\theta_{j})}{\Delta \theta_{j}},$$

where *x*_*i*_(*t*; *θ*_*j *_+ Δ*θ*_*j*_) is the *i*^*th *^component of the solution of equation (5) with an increment Δ*θ*_*j *_on the *j*^*th *^parameter. The function *x*_*i*_(*t*; *θ*_*j *_+ Δ*θ*_*j*_) can be expanded into a Taylor series as follows:

(16) $$x_{i}(t;\theta_{j}+\Delta \theta_{j})=x_{i}(t;\theta_{j})+\frac{\partial x_{i}(t;\theta_{j})}{\partial \theta_{j}}\Delta \theta_{j}+\frac{{+\partial }^{2}x_{i}(t;\theta_{j}\xi \Delta \theta_{j})}{\partial \theta_{j}^{2}}\frac{\Delta \theta_{j}^{2}}{2},$$

where 0 <*ξ *< 1. If Δ*θ*_*j *_is sufficiently small, the last term of equation (16) can be truncated, leading to a linear approximation of *x*_*i*_(*t*; *θ*_*j *_+ Δ*θ*_*j*_). Substituting the linear approximation of *x*_*i*_(*t*; *θ*_*j *_+ Δ*θ*_*j*_) into equation (15) leads to

$$s(x_{i},\theta_{j})=\frac{\partial x_{i}(t;\theta_{j})}{\partial \theta_{j}}.$$

This is defined as the first-order local sensitivity of *x*_*i *_with respect to *θ*_*j *_[33]. The relative parameter sensitivity *S*(*x*_*i*_, *θ*_*j*_) of *x*_*i *_with respect to *θ*_*j *_is defined as

$$S(x_{i},\theta_{j})=\frac{\partial \ln x_{i}(t)}{\partial \ln\theta_{j}}-\frac{\theta_{j}}{x_{i}}s(x_{i},\theta_{j}).$$

Similar to the parameter sensitivity, the absolute log gain *l*(*x*_*i*_, *y*_*j*_) and log gain *L*(*x*_*i*_, *y*_*j*_) of *x*_*i *_∈ **x **with respect to *y*_*j *_∈ **y **are expressed respectively as follows:

$$\begin{array}{c} l(x_{i},y_{j})=\frac{\partial x_{i}(t)}{\partial y_{j}},\\ L(x_{i},y_{j})=\frac{\partial \ln x_{i}(t)}{\partial \ln y_{j}}=\frac{y_{j}}{x_{i}}l(x_{i},y_{j}). \end{array}$$

Once the local sensitivity is known, the calculation of the relative sensitivity is straightforward. So, for briefing, we limit our explanation on the absolute sensitivity only below.

The absolute dynamic sensitivity of *x*_*i *_with respect to *θ*_*j *_is given as

(17) $$\frac{\text{d}s(x_{i},\theta_{j})}{\text{d}t}=\sum_{k=1}^{n}\frac{\partial f_{i}}{\partial x_{k}}s(x_{k},\theta_{j})+\frac{\partial f_{i}}{\partial \theta_{j}},$$

where *f*_*i *_is the *i*^*th *^element of **f **[34]. The absolute dynamic log gain of *x*_*i *_with respect to *y*_*j *_is similar to equation (17) by replacing *θ*_*j*_, *s*(*x*_*i*_, *θ*_*j*_) with *y*_*j*_, *l*(*x*_*i*_, *y*_*j*_) respectively when *y*_*j*_(*t*) is a constant. In the case which *y*_*j*_(*t*) is a continuous time-dependent function, the whole time domain of *y*_*j*_(*t*) is partitioned into *N*_*u *_time intervals (*t*_*k*-1_, *t*_*k*_), *k *= 1,..., *N*_*u*_. Function *y*_*j*_(*t*) is parameterized by the piecewise constant functions *ω*_*k*_(*t*), *k *= 1,..., *N*_*u*_, as follows:

(18) $$y_{j}(t)\approx \sum_{k=1}^{N_{u}}u_{k}\omega_{k}(t),$$

where *u*_*k*_, *k *= 1,..., *N*_*u*_, are constant input control parameters and

$$\omega_{k}(t)=\{\begin{array}{ll} 1, & t_{k-1}\leq t<t_{k}\\ 0, & \text{otherwise}. \end{array}$$

The continuous time-dependent function *y*_*j*_(*t*) is approximated by equation (18).  The dynamic log gain *l*(*x*_*i*_, *y*_*j*_) of infinite dimension is transferred into *N*_*u *_dynamic log gains of dimension one with respect to the input control parameters, and expressed as.

$$\frac{\text{d}l(x_{i},u_{j})}{\text{d}t}=\{\begin{array}{ll} \sum_{k=1}^{n}\frac{\partial f_{i}}{\partial x_{k}}l(x_{k},u_{j})+\frac{\partial f_{i}}{\partial u_{j}}, & t_{j-1}\leq t<t_{j}\\ \sum_{k=1}^{n}\frac{\partial f_{i}}{\partial x_{k}}l(x_{k},u_{j}), & \text{otherwise}, \end{array}$$

where *j *= 1,..., *N*_*u*_.

We extended the proposed algorithm AMCM to compute the dynamic sensitivities. The dynamic sensitivities with respect to parameters (include the initial conditions) and absolute dynamic log gains can be computed simultaneously. Let **u **be the vector of input control parameters and **r **be an *N*_*r *_dimensional vector of constants which contains constant independent variables in **y **and the input control parameters in **u**. When all components of **y **are constant, the vector **r **is equal to **y**. Let **z **be an *n *+ *p *+ *N*_*r *_dimensional vector which contains model parameters *θ*, initial conditions of dependent variables **x**_0 _and constant input in **r**.  **Φ **indicates a matrix of size *n *× (*n *+ *p *+ *N*_*r*_) which contains the absolute sensitivities with respect to model parameters and initial conditions, and the absolute log gains with respect to constant independent variables and input control parameters. **z **and **Φ **have the following form:

$$\begin{array}{c} z=\left[\begin{array}{c} \theta \\ x_{0}\\ r \end{array}\right],\\ \Phi (t)=\frac{\partial x(t)}{\partial z}=\left[\frac{\partial x(t)}{\partial \theta },\frac{\partial x(t)}{\partial x_{0}},\frac{\partial x(t)}{\partial r}\right]. \end{array}$$

The model equations are rewritten as

$$\frac{\text{d}x}{\text{d}t}=f(x(t),r;\theta ),x(0)=x_{0}.$$

The sensitivity equations in matrix form can be derived by applying the chain rule to the derivative of **Φ**:

(19) $$\begin{array}{c} \frac{\text{d}\Phi }{\text{d}t}=\frac{\partial f}{\partial x}\Phi (t)+\frac{\partial f}{\partial z},\\ \Phi (0)=\left[0_{n\times p},I_{n\times n},0_{n\times N_{r}}\right]. \end{array}$$

Let *ϕ*_*i *_be the *i*^*th *^column vector of **Φ **and *z*_*i *_be the *i*^*th *^element of **z**. The matrix sensitivity equations in equation (19) are rearranged into a vector of linear ODEs:

(20) $$\begin{array}{c} \frac{\text{d}\phi }{\text{d}t}=M\phi (t)+w,\\ \phi (0)=\left[\begin{array}{c} \phi_{1}(0)\\ \phi_{2}(0)\\ \vdots \\ \phi_{n+p+N_{r}(0)} \end{array}\right], \end{array}$$

Where

$$\begin{array}{c} \phi ={\left[\begin{array}{c} \phi_{1}\\ \phi_{2}\\ \vdots \\ \phi_{n+p+N_{r}} \end{array}\right]}_{n(n+p+N_{r})\times 1},\\ w={\left[\begin{array}{c} \frac{\partial f}{\partial z_{1}}\\ \frac{\partial f}{\partial z_{2}}\\ \vdots \\ \frac{\partial f}{\partial z_{n+p+N_{r}}} \end{array}\right]}_{n(n+p+N_{r})\times 1},\\ M={\left[\begin{array}{ccc} \frac{\partial f}{\partial x} & 0 & 0\\ 0 & \ddots & 0\\ 0 & 0 & \frac{\partial f}{\partial x} \end{array}\right]}_{n(n+p+N_{r})\times n(n+p+N_{r})}. \end{array}$$

In order to find the solution of equation (20), the whole time domain is divided into a number of non-overlapping time intervals [*t*_*i*-1_, *t*_*i*_], *i *= 1,..., *k.  *The sensitivity equations in equation (20) are transformed to algebraic equations using modified collocation method with piecewise linear polynomials as shape functions for each subinterval [*t*_*j*-1_, *t*_*j*_], *t*_*i*-1 _≤ *t*_*j*-1 _<*t*_*j *_≤ *t*_*i*_:

(21) $$\phi (t_{j})=\phi (t_{j-1})+\frac{1}{2}\eta_{j}\{M(t_{j})\phi (t_{j})+w(t_{j})+M(t_{j-1})\phi (t_{j-1})+w(t_{j-1})\},$$

where *η*_*j *_is the step size in time *t*_*j*_. This equation can be rewritten as:

(22) $$\phi (t_{j})=\frac{1}{2}\eta_{j}M(t_{j})\phi (t_{j})+c=h(\phi (t_{j})),$$

where the constant vector

$$c=\phi (t_{j-1})+\frac{1}{2}\eta_{j}\{w(t_{j})+M(t_{j-1})\phi (t_{j-1})+w(t_{j-1})\}.$$

The value of **w**(*t*_*j*_) can be evaluated if the values of **x**(*t*_*j*_) have been computed when solving equation (8) by the ODE solver. The value of the **M**(*t*_*j*_) in equation (22) must be calculated before the solution of equation (21) can be obtained by an iteration method. When the value of the Jacobian matrix ∂**f**/∂**x **in time *t*_*j *_is known, the value of the **M**(*t*_*j*_) can be obtained straightforwardly. There is no requirement for computing the value of the Jacobian matrix here due to it has been computed for step size control when solving equation (8) by the ODE solver.

Equation (22) is similar to equation (10), we can apply the fixed point theorem to *ϕ *= **h**(*ϕ*) to get the maximum *η*_*j *_satisfying the following condition

$${\left\|\frac{\partial h(\phi )}{\partial \phi }\right\|}_{p}=\frac{1}{2}\eta_{j}||M(t_{j})|{|}_{p}<1.$$

According to the definition of the matrix norm, it is easy to verify that ||**M**||_*p *_= ||∂**f**/∂**x**||_*p *_and we have

(23) $${\left\|\frac{\partial h(\phi )}{\partial \phi }\right\|}_{p}=\frac{1}{2}\eta_{j}{\left\|\frac{\partial f(x,r;\theta )}{\partial x}\right\|}_{p}<1.$$

Equation (23) is the same as equation (14), so the same criterion is used to determine the step size *η*_*j *_for computing the time course and sensitivity profile with the guarantee of convergence and existence of a fixed point. The dynamic sensitivities can be obtained directly by solving equation (21) after the time profile of **x **has been computed and the step size *η*_*j *_has been decided by the ODE solver.

## Competing interests

The authors declare that they have no competing interests.

## Authors' contributions

WHW developed and implemented the algorithm and drafted the manuscript. FSW conceived of the study, participated in its design and coordination, and helped to draft the manuscript. MSC assisted in developing the algorithm and finalizing the manuscript. All authors read and approved the final manuscript.
